# Supplementary material for: Defence-mediated phloem restriction of a plant virus facilitates insect transmission
Source: Nat Commun. 2025 Dec 23;17:152. doi: 10.1038/s41467-025-67827-w (PMC12775506; doi:10.1038/s41467-025-67827-w)
Supplement: Supplementary file 1 — Supplementary Information [file 41467_2025_67827_MOESM1_ESM.pdf]

Supplementary Tables 1-3 and Figures 1-13

Supplementary Table 1. Natural hosts of TbCSV isolates in Yunnan province.

Sample No*.	Year	Location	Number of C4 variants	Host	Accession number
Y41	2001	Yunnan	Two	*Solanum lycopersicum*	AJ457986
YN18	2008	Yunnan	Two	*Mirabilis jalapa*	GU199584
YN20	2008	Yunnan	Two	*Alternanthera philoxeroides*	GU199583
YN3702	2013	Yunnan	Two	*Nicotiana tabacum*	MN481122
Y3560	2014	Yunnan	Two	*Citrullus lanatus*	KU198364
YN5868	2016	Yunnan	Two	*Dadura stramonium*	
YN6312	2017	Yunnan	Two	*Malvastrum coromandelianum*	MN481179
Y1	1999	Yunnan	One	*Nicotiana tabacum*	AF240675
Y35	2001	Yunnan	One	*Nicotiana tabacum*	AJ420318
YN1277	2010	Yunnan	One	*Solanum lycopersicum*	
YN2247	2012	Yunnan	One	*Solanum lycopersicum*	KX290925
YN4501	2014	Yunnan	One	*Solanum lycopersicum*	KU934094
YN4543	2014	Yunnan	One	*Solanum lycopersicum*	MN481133
YN5228	2015	Yunnan	One	*Solanum lycopersicum*	MN481153
YN5747	2016	Yunnan	One	*Capsicum annuum*	MN481159
YN6297	2017	Yunnan	One	*Solanum lycopersicum*	MN481172
YN6317	2017	Yunnan	One	*Solanum lycopersicum*	MN481181
YN6318	2017	Yunnan	One	*Solanum lycopersicum*	MN481182

*YN or Y indicate the samples collected from Yunnan province.

Supplementary Table 2. Geographical distribution of TbCSV isolates from tomato field samples in Yunnan province.

TbCSV isolate	Year	Location	Number of C4 variants	Host	Accession number
YN1277	2010	Yunnan, Dehong	One	*Solanum lycopersicum*	
YN2244	2012	Yunnan, Yuxi	Two	*Solanum lycopersicum*	
YN2247	2012	Yunnan, Yuxi	One	*Solanum lycopersicum*	KX290925
YN3343	2013	Yunnan, Honghe	Two	*Solanum lycopersicum*	KU934098
YN4501	2014	Yunnan, Chuxiong	One	*Solanum lycopersicum*	KU934094
YN4519	2014	Yunnan, Chuxiong	Two	*Solanum lycopersicum*	KU934095
YN4524	2014	Yunnan, Chuxiong	One	*Solanum lycopersicum*	KU934096
YN4530	2014	Yunnan, Chuxiong	Two	*Solanum lycopersicum*	KU934097
YN4531	2014	Yunnan, Chuxiong	Two	*Solanum lycopersicum*	MN481128
YN4538	2014	Yunnan, Chuxiong	One	*Solanum lycopersicum*	MN481158
YN4543	2014	Yunnan, Chuxiong	One	*Solanum lycopersicum*	MN481133
YN4958	2015	Yunnan, Dehong	Two	*Solanum lycopersicum*	
YN5228	2015	Yunnan, Chuxiong	One	*Solanum lycopersicum*	MN481153
YN5438	2016	Yunnan, Honghe	One	*Solanum lycopersicum*	MN481157
YN6297	2017	Yunnan, Honghe	One	*Solanum lycopersicum*	MN481172
YN6317	2017	Yunnan, Honghe	One	*Solanum lycopersicum*	MN481181
YN6318	2017	Yunnan, Honghe	One	*Solanum lycopersicum*	MN481182

*YN indicates that the samples were collected from Yunnan province, China.

Supplementary Table 3. Primers used in this study.

Primers	Sequence
pGD-GFP-NbPEN3-F-SalI	TCAAGCTTCGAATTCTGCAGTCGACATGGAGGCAAATGGGG GACCGAGAAAGG
pGD-GFP-NbPEN3-R-BamHI	ATCAGTTATCTAGATCCGGTGGATCCCTATCTAGTTTGGAAG TTCAATGTC
pCambia-TbCSV(Y35)C4-Flag-F-AscI	GACTCTAGAGGATCTCGAGGCGCGCCATGGGTCTCCTCACC TGCATG
pCambia-TbCSV(Y41)C4-Flag-F-AscI	GACTCTAGAGGATCTCGAGGCGCGCCATGAAGATGGGACTC CTCACCTGCATG
pCambia-TbCSV(Y35-2C4)C4-Flag-F-AscI	GACTCTAGAGGATCTCGAGGCGCGCCATGAAGATGGGTCTC CTCACCTGCATG
pCambia-TbCSV Y35 C4-Flag-R-SalI	TCGTCCTTGTAGTCCATGTCGACATATATTGAGGGCCGCTG CTTTGG
pCambia-TbCSV Y41 C4-Flag-R-SalI	TCGTCCTTGTAGTCCATGTCGACGTATATTGAGGGCCGAAG CTT
NbPEN3-qPCR-F	ACTGGTGCATTTAGGCCAGGAGTATTG
NbPEN3-qPCR-R	GTGCAGACAACAGTTCTCCCGGT
p2YN-TbCSV Y35 C4-F-PacI	TTAATTAACATGGGTCTCCTCACCTGCATG
p2YN-TbCSV Y35-2C4 C4-F-PacI	TTAATTAACATGAAGATGGGTCTCCTCACCTGCATG
p2YN-TbCSV Y35 C4-R-SpeI	ACTAGTATATATTGAGGGCCGCTGCTTTGG
p2YN-TbCSV Y41 C4-F-PacI	TTAATTAACATGAAGATGGGACTCCTCACCTGCATG
p2YN-TbCSV Y41 C4-R-SpeI	ACTAGTGTATATTGAGGGCCGAAGCTT
p2YC-NbCAS-F-PacI	TTAATTAACATGGCGCTTAGAGCTTCAGCCAC
p2YC-NbCAS-R-SpeI	ACTAGTATCACTACCCCCTGAAAGCAATTTG
pGADT7-NbCAS-F-EcoRI	ATATGGCCATGGAGGCCAGTGAATTCATGGCGCTTAGAGCT TCAGCCAC
pGADT7-NbCAS-R-BamHI	TGCAGCTCGAGCTCGATGGATCCATCACTACCCCCTGAAAG CAATTTG
pGBKT7-TbCSV Y41 C4-F-EcoRI	CATATGGCCATGGAGGCCGAATTCATGAAGATGGGACTCCT CACCTGCATG
pGADT7-TbCSV Y41 C4-R-BamHI	GGCCGCTGCAGGTCGACGGATCCGTATATTGAGGGCCGAA GCTT
NbPR1-qPCR-F	ACAAGACTATTTGGATGCCC
NbPR1-qPCR-R	GCCGTATTGACCATGAGAATG
Nbactin-qPCR-F	CAATCCAGACACTGTACTTTCTCTC
Nbactin-qPCR-R	AAGCTGCAGGTATCCATGAGACTA

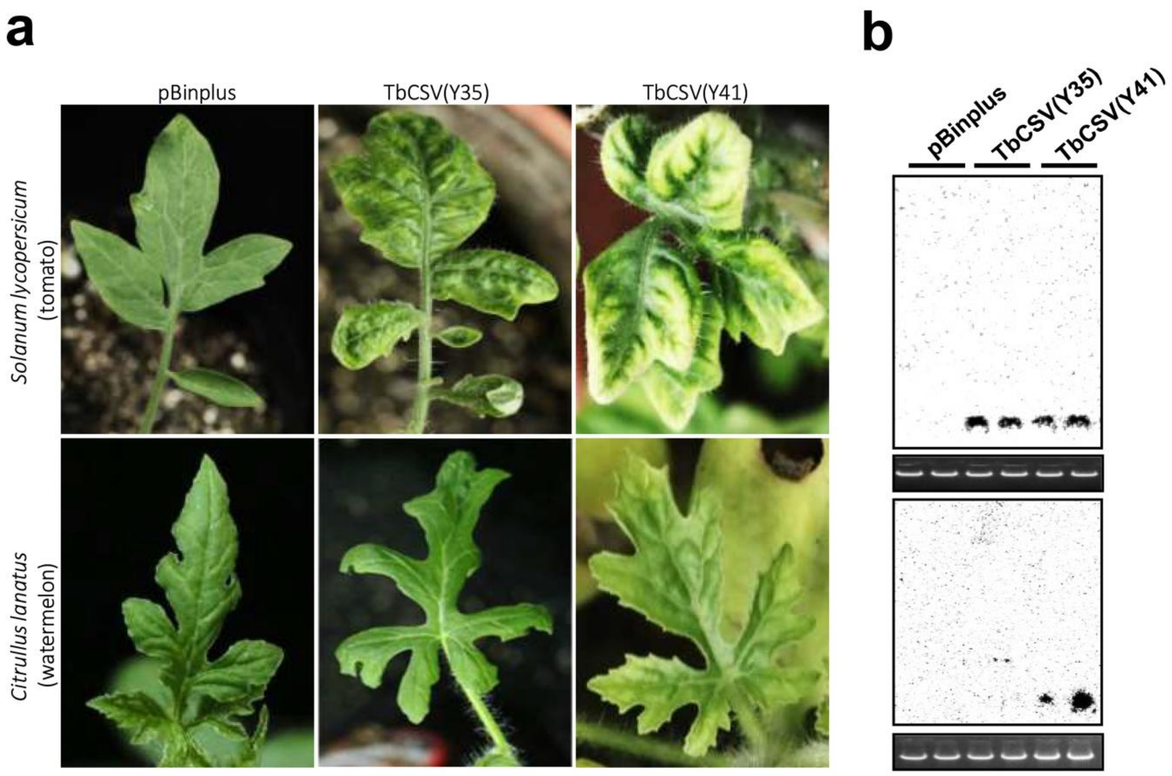

Supplementary Fig. 1. The additional C4 variant encoded by TbCSV(Y41) widens host range. a, TbCSV(Y41), expressing two C4 variants, has a wider host range compared to TbCVS(Y35). Symptoms caused by TbCSV(Y41) and TbCSV(Y35) in tomato and watermelon plants at 21 days post-inoculation (dpi). **b,** Southern blot analysis of TbCSV(Y35) and TbCSV(Y41) accumulation in tomato and watermelon plants at 21 dpi. Southern blot was hybridized with a *TbCSV CP* probe. Total nucleic acids (50 µg DNA) were extracted from inoculated tomato and watermelon plants at 21 dpi. Total genomic DNA visualized by ethidium bromide staining is shown as loading control. Experiments in (**b**) were repeated three times with similar results.

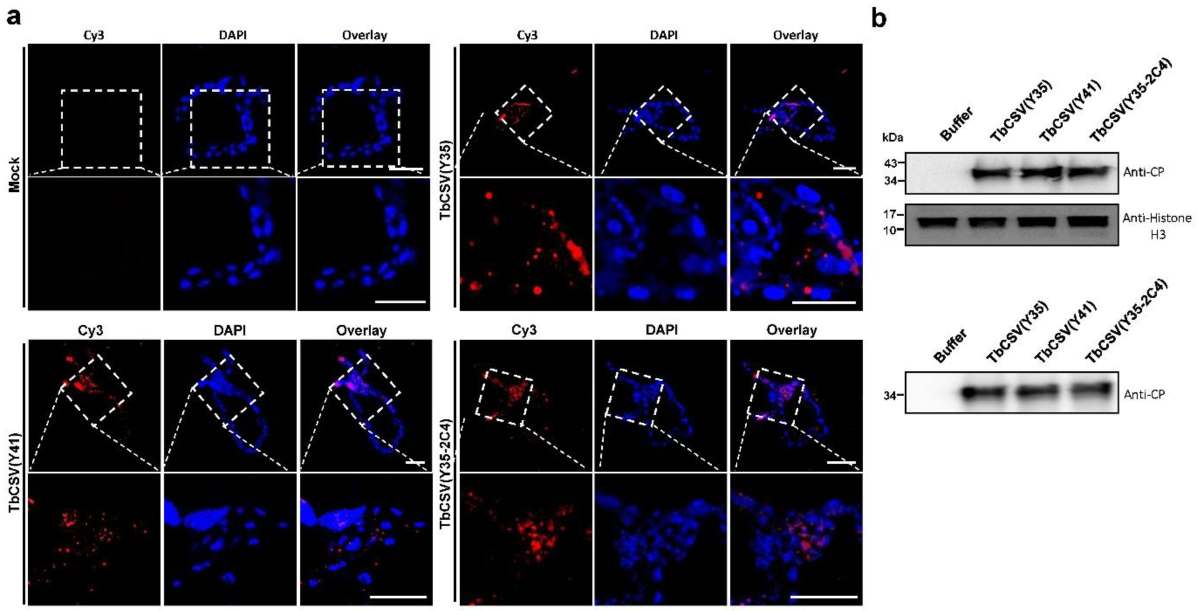

Supplementary Fig. 2. TbCSV(Y41) and TbCSV(Y35), encoding a different number of C4 protein variants, have similar capacity to overcome the midgut barrier in the insect vector. a, Immunofluorescence detection of TbCSV in midguts of Zhejiang II whiteflies after they feeding with artificial feed containing TbCSV(Y35), TbCSV(Y41), or TbCSV(Y35-2C4) virions. TbCSV CP is shown in red; nuclei are shown in blue. At least 10 midguts were examined for each treatment. Representative images are shown. White boxes indicate areas showing a magnified view. Scale bar = 100 μm. **b,** Immunoblot analysis of virus accumulation in whiteflies after feeding with feed containing TbCSV(Y35), TbCSV(Y41), or TbCSV(Y35-2C4) virions. TbCSV(Y35), TbCSV(Y41) and TbCSV(Y35-2C4) virions were extracted from virus-infected tomato plants, then added to the feed. Upper panel shows TbCSV(Y35), TbCSV(Y41), and TbCSV(Y35-2C4) accumulation in whiteflies. Histone H3 is used as loading control. Lower panel shows that the accumulation of TbCSV(Y35), TbCSV(Y41), and TbCSV(Y35-2C4) in feed. Experiments in (**a** and **b**) were repeated three times with similar results.

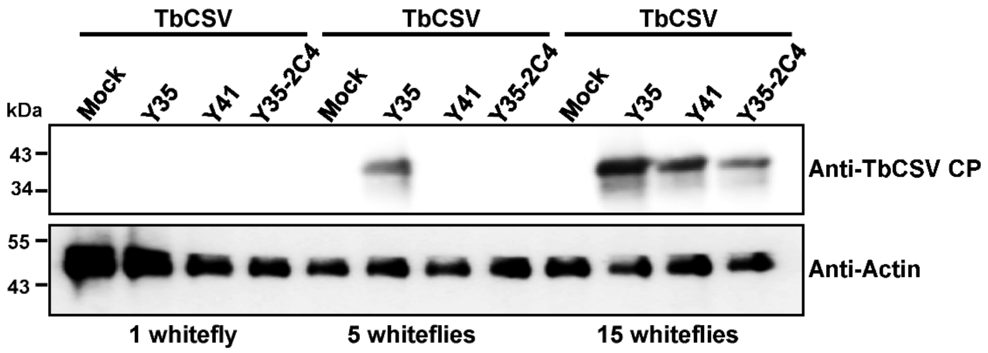

Supplementary Fig. 3. TbCSV(Y35), encoding one C4 variant only, has higher transmission efficiency. Immunoblot analysis of TbCSV(Y35), TbCSV(Y41), and TbCSV(Y35-2C4) accumulation in tomato plants inoculated with 1, 5, or 15 viruliferous whiteflies. Non-viruliferous whiteflies were used as mock treatment. TbCSV was detected using a monoclonal antibody against the coat protein (CP). Actin was used as loading control.

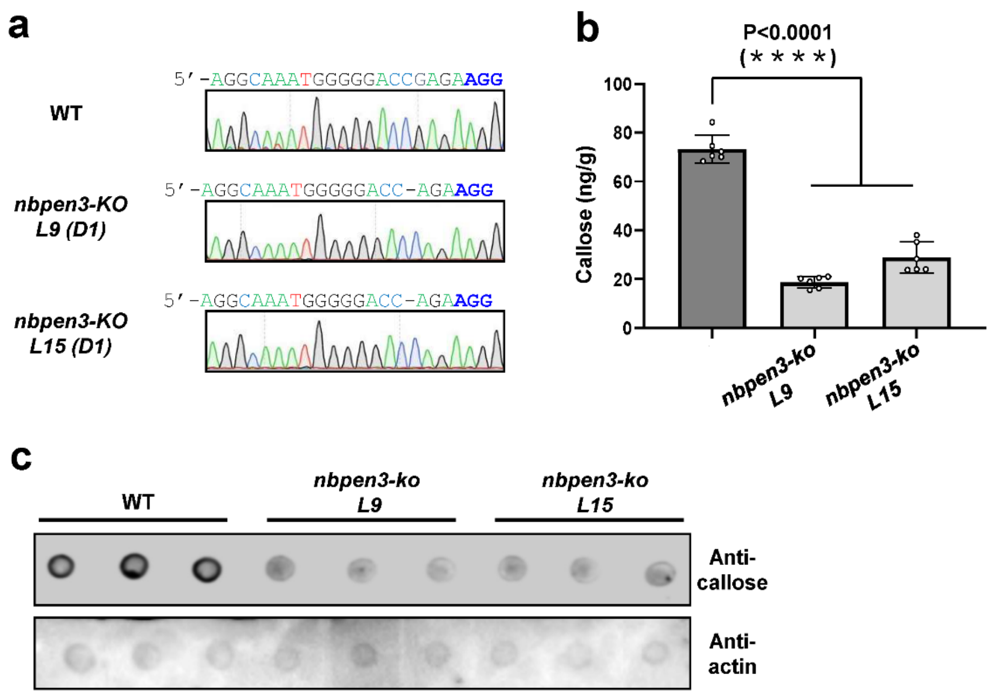

Supplementary Fig. 4. NbPEN3 is required for callose accumulation in *Nicotiana benthamiana* plants. a, Mutations identified at the *NbPEN3* locus in *N. benthamian*a plants edited by the CRISPR-Cas9 system. Blue bold letters indicate the protospacer adjacent motif (PAM). *nbpen3(D1)* represents deletion of one nucleotide in the *NbPEN3* locus. **b-c,** ELISA (**b**) and immunoblot analysis (**c**) of callose accumulation in WT and *nbpen3* knock-out *N. benthamiana* plants. ELISA data represent the mean ± standard deviation (SD). Statistical differences were analyzed by two-sided, unpaired Student's t-test (****P < 0.0001). Six individual plants per treatment were used to analyze callose accumulation. Error bar denotes the standard deviation of the mean. Individual *P* values are denoted above the comparison lines. Actin was used as loading control. Experiments in (**b** and **c**) were repeated three times with similar results.

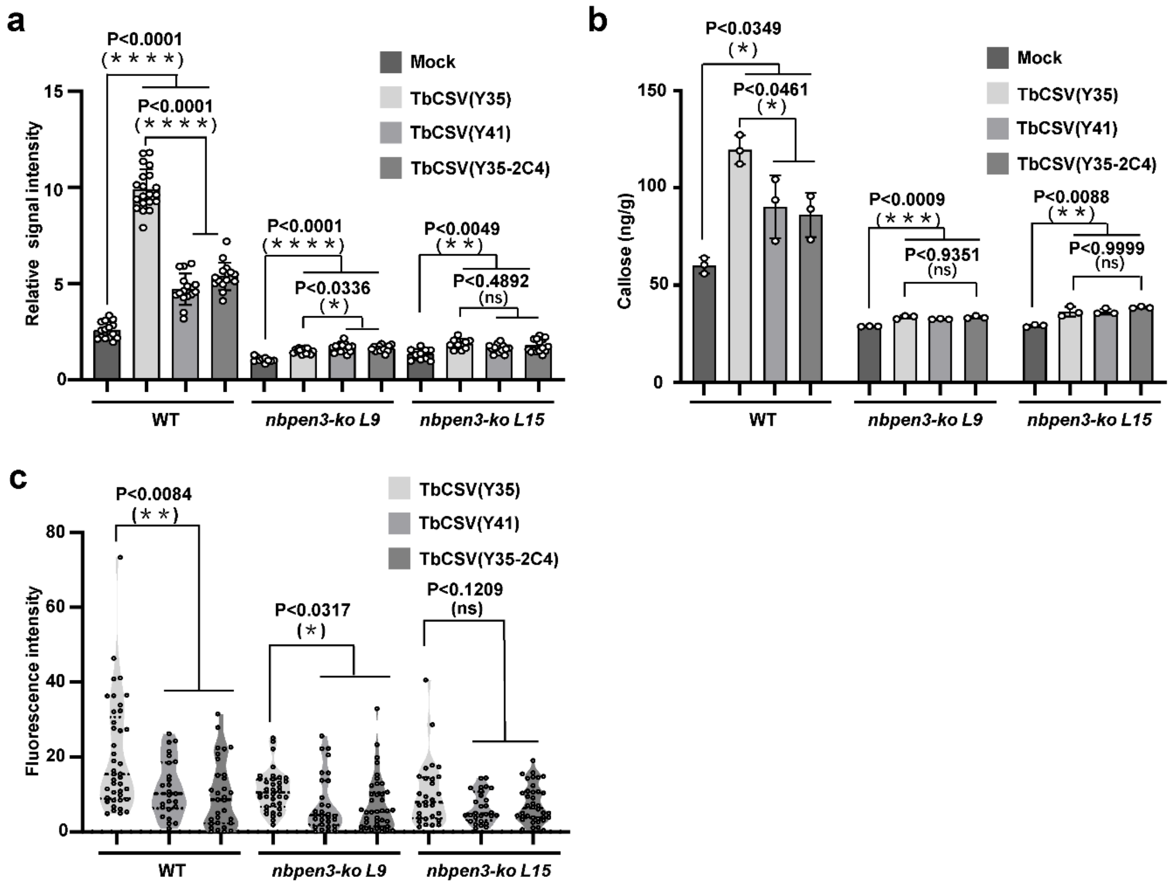

Supplementary Fig. 5. NbPEN3 is critical for callose-deposition induced by virus infection. a, Relative intensity of the aniline blue fluorescent signal in the phloem (fluorescence intensity/selected area) in wild-type (WT) and *nbpen3* knock-out *N. benthamiana* plants infected by TbCSV(Y35), TbCSV(Y41), or TbCSV(Y35-2C4), as measured by ImageJ; at least 10 areas were selected in each treatment. Statistical differences were analyzed by two-sided, unpaired Student's t-test (ns: not significant, *P < 0.05, **P < 0.01, ****P < 0.0001). Data are present as mean values ± standard deviation (SD). Individual *P* values are denoted above the comparison lines. **b,** ELISA analysis of callose accumulation in wild-type (WT) and *nbpen3* knock-out *N. benthamiana* plants infected by TbCSV(Y35), TbCSV(Y41), or TbCSV(Y35-2C4). Statistical differences were analyzed by two-sided, unpaired Student's t-test (ns: not significant, *P < 0.05, **P < 0.01, ***P < 0.001). Data are present as mean values ± SD. Individual *P* values are denoted above the comparison lines. Error bars show standard deviation for three independent

experiments. **c,** Immunofluorescence intensity of TbCSV signal in the phloem of leaf vein sections (fluorescence intensity/selected area) in wild-type (WT) and *nbpen3* knock-out *N. benthamiana* plants infected by TbCSV(Y35), TbCSV(Y41), or TbCSV(Y35-2C4), as measured by ImageJ; at least 25 areas were selected in each condition. Statistical differences were analyzed by two-sided, unpaired Student's t-test (ns: not significant, *P < 0.05, **P < 0.01). Individual *P* values are denoted above the comparison lines. The median and quartiles are shown as the dashed lines in the graph. Experiments in (**a**, **b** and **f**) were repeated three times with similar results.

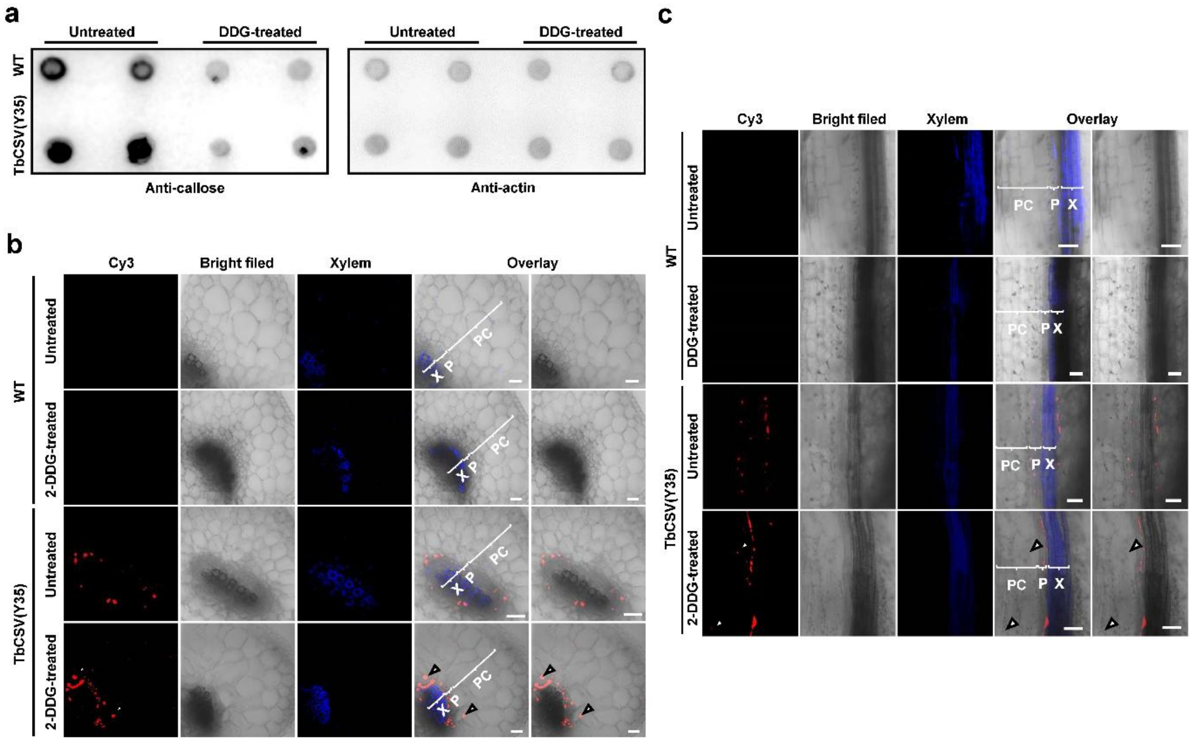

Supplementary Fig. 6. Callose deposition plays a critical role in phloem restriction of virus. a, Immunoblot analysis of callose accumulation in WT and TbCSV(Y35)-infected *Nicotiana benthamina* plants treated with ddH_2O (Mock) or 200 µM 2-deoxy-D-glucose (2-DDG). Plant tissues treated with ddH_2O or 200 µM 2-DDG for 4 days were prepared for immunoblot analysis and actin was used as loading control. **b-c,** TbCSV strain expressing only one C4 variant (Y35) overcomes phloem restriction and reach parenchyma tissue in *N. benthamiana* plants treated with 2-DDG. The distribution of TbCSV(Y35) was visualized in a cross-section (**b**) and longitudinal-section (**c**) using an antibody against TbCSV CP (red). Autofluorescence of highly lignified tissues is shown in blue. PC, parenchyma; P, phloem; X, xylem. White arrowheads indicate TbCSV in parenchyma cells. Scale bar = 50 µm. Experiments in (**a**, **b** and **c**) were repeated three times with similar results.

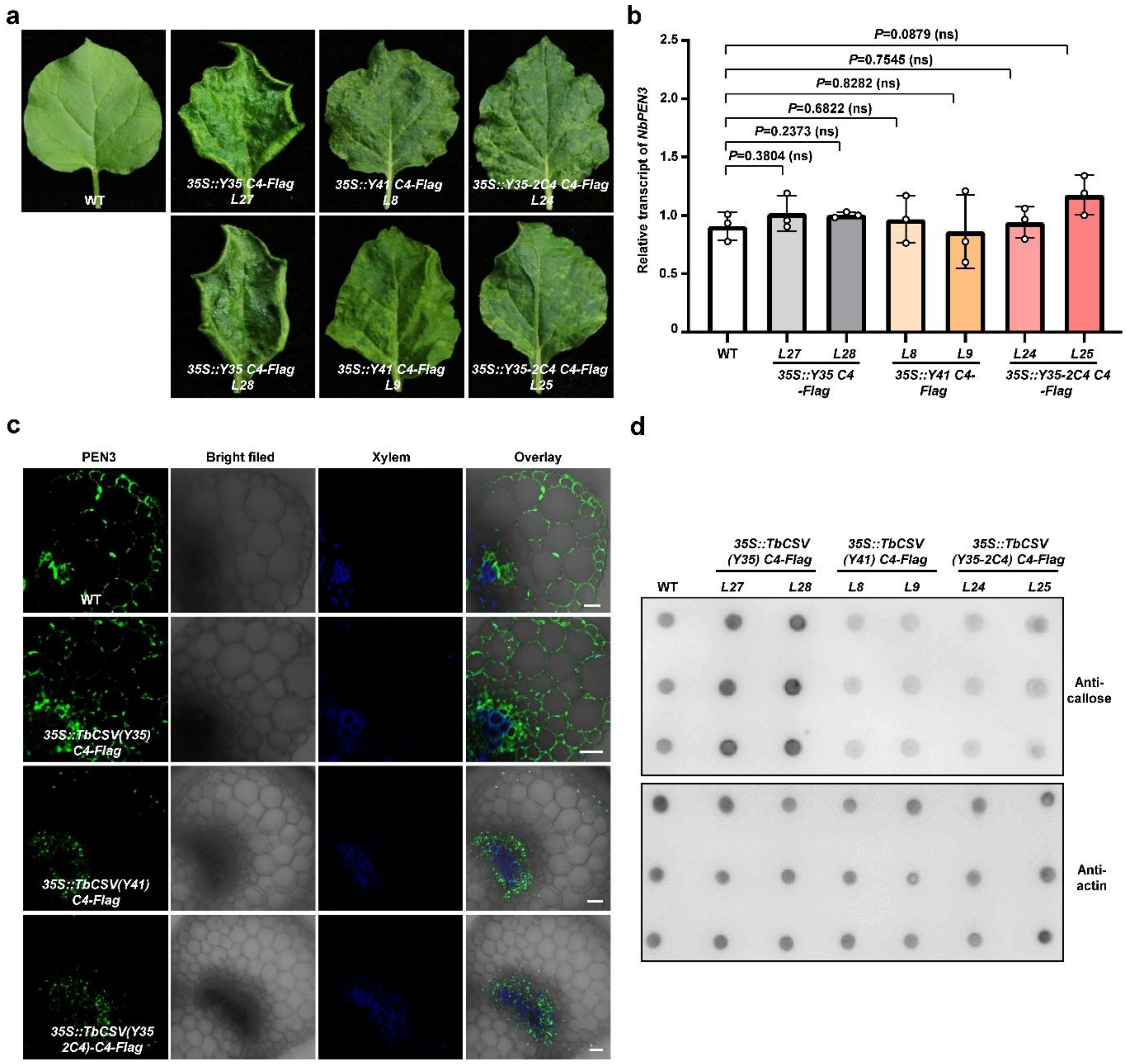

Supplementary Fig. 7. The effects of chloroplast-localized C4 on symptom development, *NbPEN3* transcription, and callose deposition. a, Phenotype of *35S::TbCSV(Y35) C4-Flag*, *35S::TbCSV(Y41) C4-Flag*, and *35S::TbCSV(Y35-2C4) C4-Flag* transgenic *N. benthamiana* plants. Upward leaf curling and abnormal shapes in leaves of two independent *35S::TbCSV(Y35) C4-Flag* transgenic *N. benthamiana* plants and mosaic phenotype in leaves of two independent *35S::TbCSV(Y41) C4-Flag*, and *35S::TbCSV(Y35-2C4) C4-Flag* transgenic *N. benthamiana* plants are shown. Photographs were taken at 50 days post-germination. **b,** Quantitative PCR analysis of *NbPEN3* relative transcript in *35S::TbCSV(Y35) C4-Flag*, *35S::TbCSV(Y41) C4-Flag*, and *35S::TbCSV(Y35-2C4) C4-Flag* transgenic *N. benthamiana* plants. Relative accumulation of *NbPEN3* transcripts is

normalized against that of *ACTIN*. Statistical differences were analyzed by two-sided, unpaired Student's t-test (ns: not significan). Data are present as mean values ± standard deviation (SD). Individual *P* values are denoted above the comparison lines. Error bar denotes the standard deviation of three biological replicates. **c,** Immunofluorescence detection of NbPEN3 in leaf vein sections of wild-type (WT), *35S::TbCSV(Y35) C4-Flag*, *35S::TbCSV(Y41) C4-Flag*, and *35S::TbCSV(Y35-2C4) C4-Flag* transgenic *N. benthamiana* plants. The distribution of NbPEN3 was visualized in a cross-section using an antibody against PEN3 (green). Autofluorescence of highly lignified tissues is shown in blue. Scale bar = 50 μm. **d,** Immunoblot analysis of callose accumulation in WT, *35S::TbCSV(Y35) C4-Flag*, *35S::TbCSV(Y41) C4-Flag*, and *35S::TbCSV(Y35-2C4) C4-Flag* transgenic *N. benthamiana* plants. Two independent lines of *35S::TbCSV(Y35) C4-Flag*, *35S::TbCSV(Y41) C4-Flag*, and *35S::TbCSV(Y35-2C4) C4-Flag* transgenic *N. benthamiana* plants were used. Actin was used as loading control. Experiments in (**b**, **c** and **d**) were repeated three times with similar results.

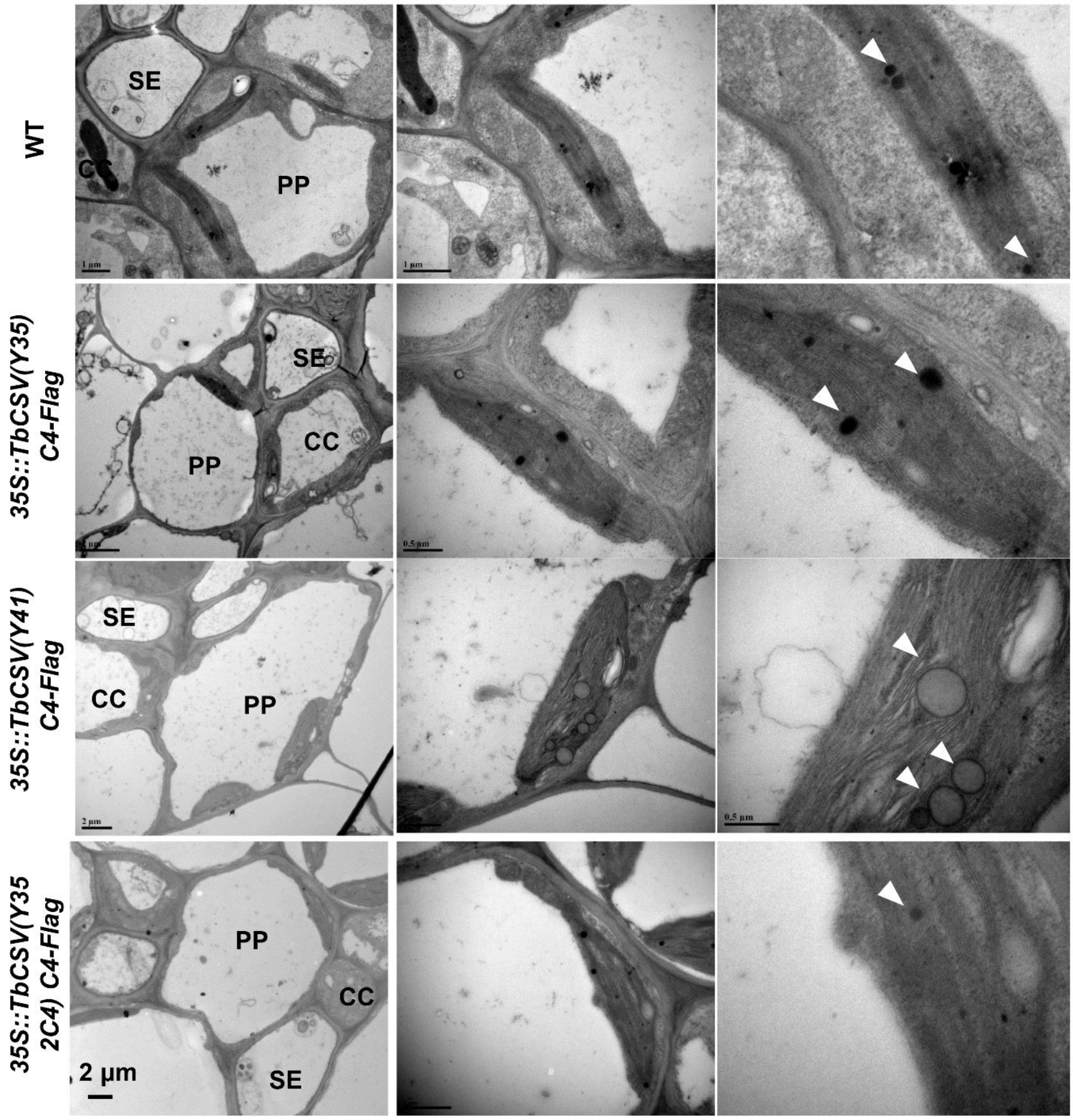

Supplementary Fig. 8. Electron microscopy of chloroplasts in phloem parenchyma of wild-type (WT), *35S::TbCSV(Y35) C4-Flag*, *35S::TbCSV(Y41) C4-Flag*, and *35S::TbCSV(Y35-2C4) C4-Flag* transgenic *N. benthamiana* plants. White arrowheads indicate osmiophilic granules. PP, phloem parenchyma; SE, sieve element; CC, companion cell.

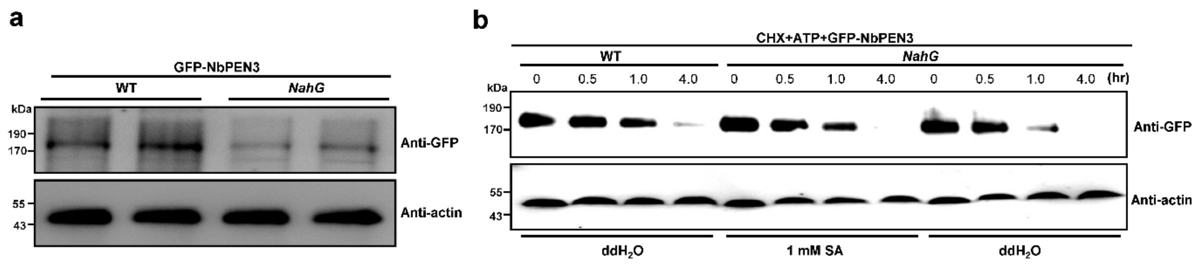

Supplementary Fig. 9. NbPEN3 stability is regulated in a SA-dependent manner. a, Immunoblotting analysis of GFP-NbPEN3 accumulation in wild-type (WT) and *NahG* transgenic *N. benthamiana* plants. GFP-NbPEN3 was detected with an anti-GFP monoclonal antibody. Actin was used as loading control. **b,** Western blot analysis of the stability of GFP-NbPEN3 in WT and *NahG* transgenic *N. benthamiana* plants treated with ddH_2O or SA by semi-*in vivo* assays. Actin was used as loading control. Experiments in (**a** and **b**) were repeated three times with similar results.

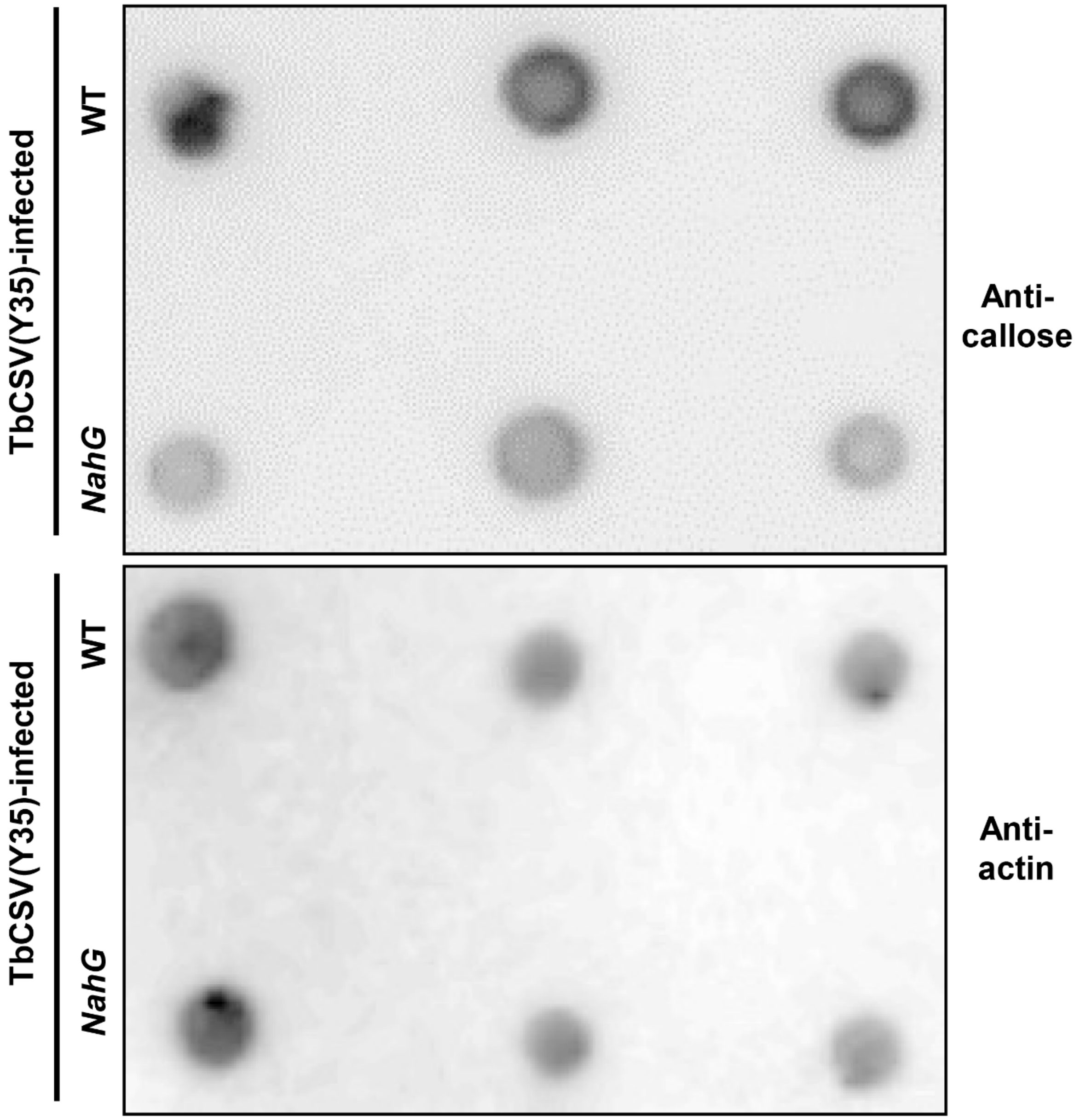

Supplementary Fig. 10 Immunoblot analysis of callose accumulation in TbCSV-infected WT and *NahG* transgenic *Nicotiana benthamiana* plants. Actin was used as loading control. Experiments were repeated three times with similar results.

5′-CCGAGGAGGTTGGGGAGTAGTAG
GGCTCCTCCAACCCCTCATCATC-5′

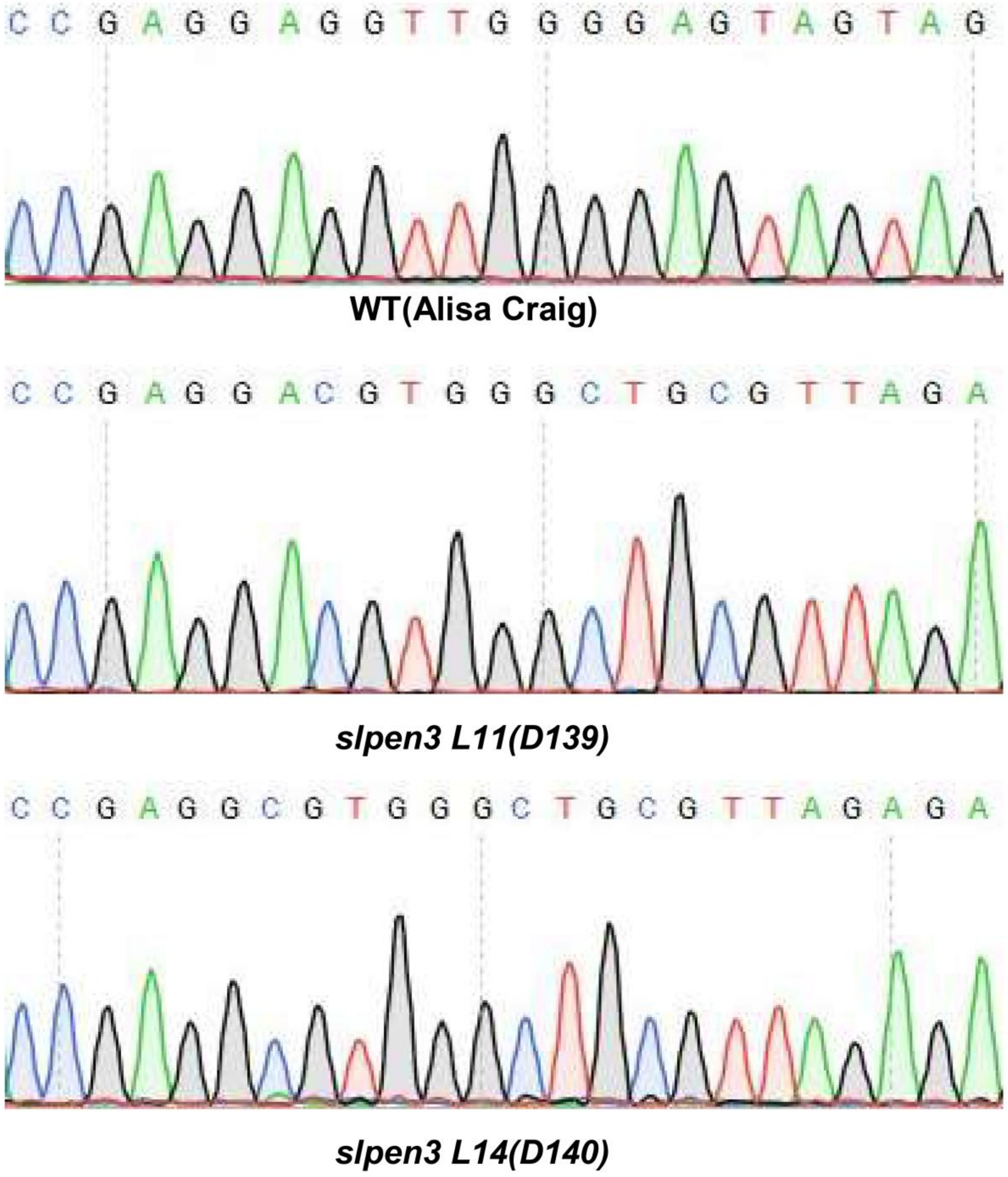

Supplementary Fig. 11. Mutation types identified at *SlPEN3* locus in *Solanum lycopersicum* plants edited by Cas9 system. Red letters indicate the protospacer adjacent motif (PAM). *slpen3(D139)* and *slpen3(D140)* represent deletion of 139 or 140 nucleotides in the *SlPEN3* locus.

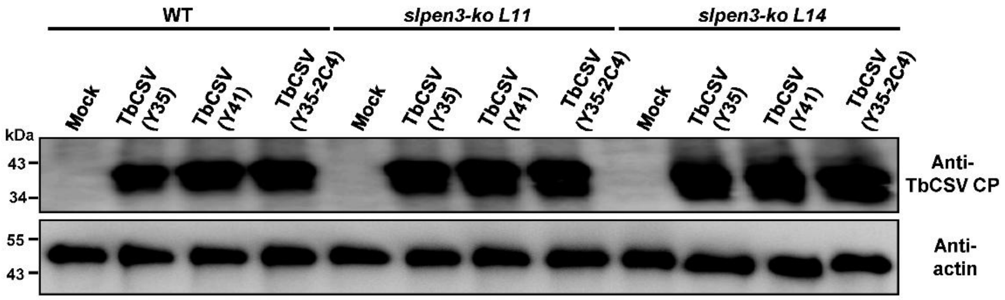

Supplementary Fig. 12. Western blot of TbCSV CP accumulation in wild-type (WT) and *SlPEN3* knock-out tomato plants infected by TbCSV(Y35), TbCSV(Y41) or TbCSV(Y35-2C4) at 30 dpi. Accumulation of actin is shown as control. Experiments were repeated three times with similar results.

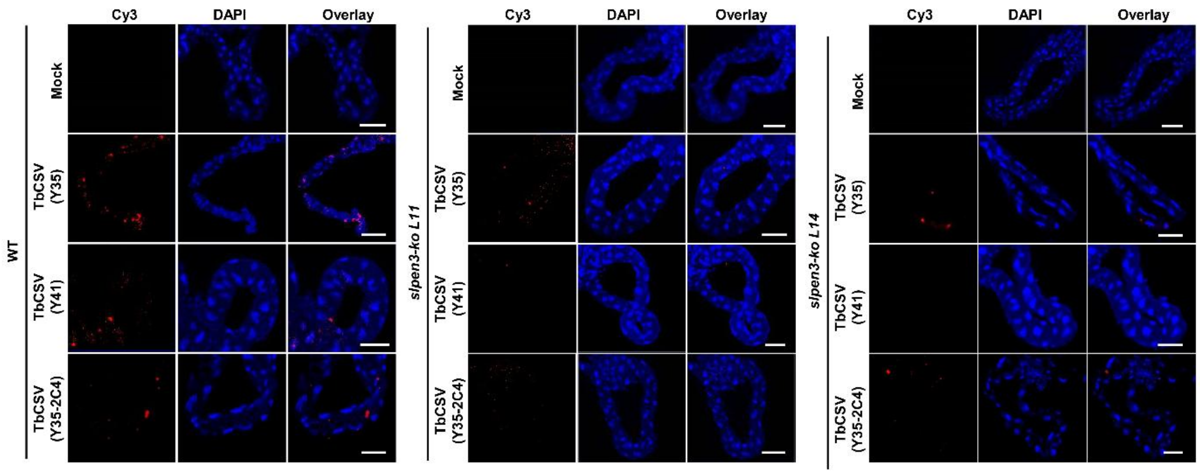

Supplementary Fig. 13. Immunofluorescence detection of TbCSV in midguts of Zhejiang II whiteflies after feeding on wild-type (WT) or *slpen3* tomato plants infected by TbCSV(Y35), TbCSV(Y41) or TbCSV(Y35-2C4). Confocal micrographs shown in Figure S13 are a magnified view of the representative images presented in Figure 5C. TbCSV was detected using a monoclonal antibody against the TbCSV coat protein (CP) followed by a commercial 549-conjugated secondary antibody (red), and nuclei were stained with DAPI (blue). Scale bar = 100 μm. Experiments were repeated three times with similar results.
