# Supplementary material for: Defence-mediated phloem restriction of a plant virus facilitates insect transmission
Source: Nat Commun. 2025 Dec 23;17:152. doi: 10.1038/s41467-025-67827-w (PMC12775506; doi:10.1038/s41467-025-67827-w)
Supplement: Supplementary file 2 — Reporting Summary [file 41467_2025_67827_MOESM2_ESM.pdf]

nature portfolio

Corresponding author(s): Xueping Zhou

Last updated by author(s): Dec 8, 2025

Reporting Summary

Nature Portfolio wishes to improve the reproducibility of the work that we publish. This form provides structure for consistency and transparency in reporting. For further information on Nature Portfolio policies, see our Editorial Policies and the Editorial Policy Checklist.

Statistics

For all statistical analyses, confirm that the following items are present in the figure legend, table legend, main text, or Methods section.

n/a	Confirmed	
☐	☒	The exact sample size (*n*) for each experimental group/condition, given as a discrete number and unit of measurement
☐	☒	A statement on whether measurements were taken from distinct samples or whether the same sample was measured repeatedly
☐	☒	The statistical test(s) used AND whether they are one- or two-sided<br>*Only common tests should be described solely by name; describe more complex techniques in the Methods section.*
☒	☐	A description of all covariates tested
☒	☐	A description of any assumptions or corrections, such as tests of normality and adjustment for multiple comparisons
☐	☒	A full description of the statistical parameters including central tendency (e.g. means) or other basic estimates (e.g. regression coefficient) AND variation (e.g. standard deviation) or associated estimates of uncertainty (e.g. confidence intervals)
☐	☒	For null hypothesis testing, the test statistic (e.g. *F*, *t*, *r*) with confidence intervals, effect sizes, degrees of freedom and *P* value noted<br>*Give P values as exact values whenever suitable.*
☒	☐	For Bayesian analysis, information on the choice of priors and Markov chain Monte Carlo settings
☒	☐	For hierarchical and complex designs, identification of the appropriate level for tests and full reporting of outcomes
☒	☐	Estimates of effect sizes (e.g. Cohen's *d*, Pearson's *r*), indicating how they were calculated

Our web collection on statistics for biologists contains articles on many of the points above.

Software and code

Policy information about availability of computer code

Data collection	All confocal images were acquired with ZEN 2012(version 1.1.13064.302) software. qRT-PCR data were acquired with Roche lightcycler 480 software v1.5. Western blot chemiluminescence signals were acquired with the Image Quant LAS (GE Healthcare).
Data analysis	Statistical analysis was performed using GraphPad Prism 8.

For manuscripts utilizing custom algorithms or software that are central to the research but not yet described in published literature, software must be made available to editors and reviewers. We strongly encourage code deposition in a community repository (e.g. GitHub). See the Nature Portfolio guidelines for submitting code & software for further information.

Data

Policy information about availability of data

All manuscripts must include a data availability statement. This statement should provide the following information, where applicable:

- Accession codes, unique identifiers, or web links for publicly available datasets
- A description of any restrictions on data availability
- For clinical datasets or third party data, please ensure that the statement adheres to our policy

Accession number of viruses and genes is available as a Supplementary Information file.

Human research participants

Policy information about studies involving human research participants and Sex and Gender in Research.

Reporting on sex and gender	No.
Population characteristics	No.
Recruitment	No.
Ethics oversight	No.

Note that full information on the approval of the study protocol must also be provided in the manuscript.

Field-specific reporting

Please select the one below that is the best fit for your research. If you are not sure, read the appropriate sections before making your selection.

[x] Life sciences [ ] Behavioural & social sciences [ ] Ecological, evolutionary & environmental sciences

For a reference copy of the document with all sections, see nature.com/documents/nr-reporting-summary-flat.pdf

Life sciences study design

All studies must disclose on these points even when the disclosure is negative.

Sample size	At least two biological replicates were performed and all data were used for the analysis in this study.
Data exclusions	No data were excluded.
Replication	Each replication was successful.
Randomization	Experimental plant materials were collected randomly without any bias.
Blinding	Blinding was not performed. Blinding was not necessary because our results are objective and do not rely on subjective judgment. When performing bioinformatic analyses, the same parameters were applied and no manual intervention was applied.

Reporting for specific materials, systems and methods

We require information from authors about some types of materials, experimental systems and methods used in many studies. Here, indicate whether each material, system or method listed is relevant to your study. If you are not sure if a list item applies to your research, read the appropriate section before selecting a response.

Materials & experimental systems

n/a	Involved in the study	
[ ]	[x]	Antibodies
[x]	[ ]	Eukaryotic cell lines
[x]	[ ]	Palaeontology and archaeology
[x]	[ ]	Animals and other organisms
[x]	[ ]	Clinical data
[x]	[ ]	Dual use research of concern

Methods

n/a	Involved in the study	
[x]	[ ]	ChIP-seq
[x]	[ ]	Flow cytometry
[x]	[ ]	MRI-based neuroimaging

Antibodies

Antibodies used

Mouse monoclonal anti-Flag M2, Sigma,Cat# F1804;
Mouse monoclonal anti-Histone H3, Huabio, Cat# M1309-1;
Mouse monoclonal antI-PEN3, PHYTOAB, Cat# PHY0177A;
HRP-conjugated anti-callose antibody, Bioroyee, Cat#RE5372;
Goat anti-mouse 549-conjugated secondary antibody, Earthox, Cat# E032410-01;
Goat anti-rabbit 488-conjugated secondary antibody, Earthox, Cat# E032220-01;
Mouse monoclonal anti-TbCSV CP, mouse monoclonal anti-TYLCV CP and mouse polyclonal anti-PLRV CP were kindly provided by Dr. Jianxiang Wu, Zhejiang University.

Validation	Commercial antibodies were validated by venders and the information can be found online.
